# Influences of RF Magnetron Sputtering Power and Gas Flow Rate on a High Conductivity and Low Drift Rate of Tungsten-Rhenium Thin-Film Thermocouples

**DOI:** 10.3390/nano12071120

**Published:** 2022-03-28

**Authors:** Zhongkai Zhang, Bian Tian, Gong Cheng, Zhaojun Liu, Jiangjiang Liu, Bingfei Zhang, Jiaming Lei, Na Zhao, Feng Han, Xudong Fang, Hao Sun, Libo Zhao

**Affiliations:** 1 State Key Laboratory for Mechanical Manufacturing Systems Engineering, Institute of Precision Engineering, School of Mechanical Engineering, Xi’an Jiaotong University, Xi’an 710049, China; zhangzk@xjtu.edu.cn (Z.Z.); t.b12@mail.xjtu.edu.cn (B.T.); cg19970417@stu.xjtu.edu.cn (G.C.); lzj2018@stu.xjtu.edu.cn (Z.L.); super_l@stu.xjtu.edu.cn (J.L.); bingfeizhang01@stu.xjtu.edu.cn (B.Z.); 3120101279@stu.xjtu.edu.cn (J.L.); hanfeng_20625@xjtu.edu.cn (F.H.); dongfangshuo30@xjtu.edu.cn (X.F.); xjtusunhao@xjtu.edu.cn (H.S.); libozhao@mail.xjtu.edu.cn (L.Z.); 2 Institute of Materials in Electrical Engineering 1, Department of Micro- and Nano Electronics, Faculty of Electical Engineering and Information Technology, RWTH Aachen University, 52074 Aachen, Germany; 3 Collaborative Innovation Center of Suzhou Nano Science and Technology, Suzhou 215123, China

**Keywords:** sensor, stability, thermoelectricity

## Abstract

Thin-Film Thermocouples (TFTCs) are characterized by their high spatial resolutions, low cost, high efficiency and low interference on the air flow. However, the thermal stability of TFTCs should be further improved for application since their accuracy is influenced by joule heat and temperature time drift. In this paper, 3D molecular dynamics and finite element analysis are used for structural design. The effects of RF magnetron sputtering power and gas flow rate on conductivity and temperature time drift rate (DT) of high thermal stability tungsten–rhenium (95% W/5% Re vs. 74% W/26% Re) TFTCs were analyzed. According to the experimental results, the average Seebeck coefficient reached 31.1 µV/°C at 900 °C temperature difference (hot junction 1040 °C) with a repeatability error at ±1.37% in 33 h. The conductivity is 17.1 S/m, which is approximately 15.2 times larger than the compared tungsten-rhenium sample we presented, and the DT is 0.92 °C/h (1040 °C for 5 h), which is 9.5% of the old type we presented and 4.5% of compared ITO sample. The lumped capacity method test shows that the response time is 11.5 ms at 300 °C. This indicated an important significance in real-time temperature measurement for narrow spaces, such as the aero-engine combustion chamber.

## 1. Introduction

Temperature data have been used to represent conditions in service monitoring and parameter calculation for gas turbine structure design. As the high thrust-to-weight ratio aero engine advances, the service temperature and duration of the combustion chamber of gas turbines operating in harsh environments have been steadily increasing [1,2]. To monitor the performance of component materials in gas turbines, various thermal sensors (e.g., thermocouples, optical pyrometers, acoustic pyrometers, and thermal resistance sensors) have been developed.

Thin-film thermocouples (TFTCs) are a novel type of microsensor based on micro electromechanical system (MEMS) technology. They are made of highly thermally sensitive materials. The thermocouple, as it is commonly known, consists of two distinct electrodes. When there is a temperature difference between the hot and cold junctions, the direct-current (DC) voltage between them can be measured, indicating that the total output voltage is primarily determined by the material’s Seebeck coefficient and temperature difference. TFTCs are fabricated directly on structural surfaces using thin film deposition technology (e.g., an insulating layer and a device layer) and are used to measure the surface temperature in real time in situ [3,4,5]. TFTCs are thin passive thermoelectric temperature sensors that can be deposited directly on the surfaces of aeronautical engine components. They feature a high spatial resolution, a low cost, a high level of efficiency, and rapid response times [6,7]. Additionally, the integrated TFTC, due to its negligible mass, has a negligible effect on air flow [8,9,10]. In comparison to other types of thermal sensors, TFTCs have been identified as a promising thermal sensor for aeroengines. For example, optical fiber will obstruct the flow field. Real-time thermal-paint measurements are not possible. The materials used to make TFTCs for aeroengine applications should have a high thermal stability, a high melting point, and a relatively large Seebeck coefficient. TFTCs composed of platinum and rhodium (Pt/Rh) reach a maximum temperature of 1100 °C [11]. Wang Qiang [12] prepared TFTCs on aluminum oxide (Al_2_O_3_) in 2016 using titanium (Ti) and chromium (Cr) as transition layers. When the transition layer was added, the thermoelectric service performance improved. Rhodium, on the other hand, was oxidized at 800 °C, and platinum oxide volatilization of Pt/Rh TFTCs increased with increasing oxygen partial pressure [13,14]. Indium tin oxide (ITO) has been used to fabricate well-known TFTCs that operate at temperatures below 1100 °C [15], while ITO melts at temperatures around 1300 °C [16]. However, to meet the requirements, the working temperature should be increased further.

Tungsten-rhenium TFTCs exhibit superior performance due to their high melting point of approximately 2800 °C and ease of oxidation [17]. Although we previously demonstrated that high temperature failure can be avoided using an alumina protective layer on tungsten-rhenium TFTCs, the drift rate (DT) is high (9.6 °C/h), resulting in low thermoelectric stability [18]. In fact, how to maintain good time stability of thin-film thermocouple in high temperature section is a difficult problem restricting its engineering application. In this article, 3D molecular dynamics and finite element analysis (FEA) were used to design structural elements. The effects of RF magnetron sputtering power and gas flow rate on tungsten-rhenium TFTCs with high conductivity and low DT were demonstrated. The average Seebeck coefficient was 31.1 µV/°C at a temperature difference of 900 °C (hot junction 1040 °C), with a repeatability error of ± 1.37% over a 33-h period. The conductivity of tungsten-rhenium TFTCs was 17.1 S/m (15.27 times that of the compared sample), and they demonstrated the highest thermoelectric stability with a DT of 0.92 °C/h (9.5% of the old sample we presented, 4.5% of ITO TFTCs) during 5 h of service at 1040 °C. The lumped capacity method demonstrated an 11.5 ms response time at 300 °C. This high conductivity and low drift rate TFTCs at high temperature indicated an important significance in real-time temperature measurement for narrow spaces, such as the aero-engine combustion chamber.

## 2. Materials and Methods

Molecular calculation of the material system. Three-dimensional molecular dynamics analysis was used to investigate the microscopic properties of tungsten-rhenium and silicon carbide at elevated temperatures. We constructed a three-dimensional molecular dynamics simulation model of silicon carbide and tungsten-rhenium metal atomic layer. The top layer was tungsten (W), the middle layer was rhenium (Re), and the bottom layer was silicon carbide (SiC). The spacing between each atomic layer was 4 Å. By specifying the target material in the X and Y directions as periodic boundary conditions, the scale effect of the simulation could be minimized. The Verlet integration algorithm was used to calculate three-dimensional molecular dynamics, with a time step of 1 fs. Two stages were included in the simulation: relaxation and heating. The simulation would be kept at 293 K in the micro-canonical ensemble to maintain the stable state of the entire model during the relaxation stage. When the initial value of the simulation system’s temperature remains constant and the fluctuation range of the system’s pressure is equal to or close to 0 bar, the model is said to be stable. The 3D visualization software OVITO (Version 3.7.2, OVITO GmbH, Darmstadt, Germany) was used to perform final processing on the data of interactions between atomics simulated by LAMMPS (LAMMPS 3 Mar 2020, Sandia National Laboratories, New Mexico, USA), resulting in the visual snapshot. C-C, Si-Si and Si-C were calculated by three-body Tersoff potential function. The interactions between W-W, Re-Re and W-Re were described by EAM alloy potential function. Finally, the Morse potential function described the interactions between non-metallic atoms C and Si and metal atoms W and Re.

Size parameter design. The size parameters of TFTCs were designed using the stress finite element model. Thermal stress finite element analysis (FEA) using ANSYS (Version 15.0, ANSYS Inc., South Pittsburgh, Pennsylvania, USA) was used to consider the material’s isotropic, thermoplastic, and orthotropic properties.

**Figure 1:**
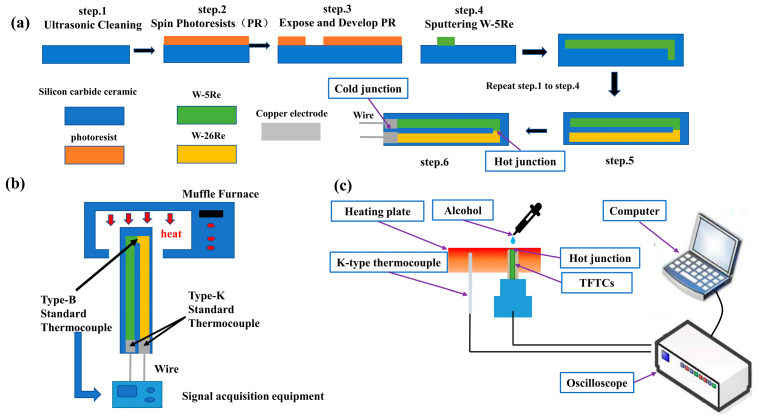
The structural and preparation process (**a**), thermoelectric characteristic test system (**b**), and response drop test system (**c**) of tungsten-rhenium thin-film thermocouples (TFTCs).

Preparation technique. TFTCs were fabricated through RF magnetron sputtering (DISCOVERY, 635, Moorestown, New Jersey, USA) from W-5Re (95%W/5%Re) and W-26Re (74%W/26%Re) high purity 101.6 mm diameter targets (purity 99.99%, ZHONGNUO Co., Beijing, China) onto 80 mm × 30 mm × 1 mm substrates after cleaning with ultrasonic with acetone, ethyl alcohol and deionized water, followed by annealing at 300 °C for 2 h. Copper (Cu, purity 99.9%, ZHONGNUO Co., Beijing, China) electrode was sputtered at the end of the cold junction of the TFTC surfaces. The film thickness was kept constant at 500 nm, while the Ar gas flow rate varied between 30 and 90 sccm and the sputtering power varied between 100 and 300 W. The sputtering parameters for nine samples are summarized in Table 1. Notably, the aluminum oxide protective layer on the surface of the sample used for high-temperature testing was also prepared according to the method previously proposed. However, the protective layer was prepared following the conductivity test. The Al_2_O_3_ coating layer above the samples was 20 nm thick. Figure 1a illustrates the structure and synthesis of tungsten-rhenium TFTCs.

Measurement technique. The tungsten-rhenium alloy wire was used to connect the TFTCs to the signal acquisition equipment (LR8431-30, HIOKI Co., Nagano, Japan). The elemental composition and conductivity of TFTCs were determined before and after temperature service using a four-probe conductivity tester (ST2258C, Suzhou Jingge Electronic Co., Suzhou, China). The TFTCs were placed in a muffle furnace (LHT 02/17/P310, Nabertherm, Lilienthal, Germany) and the temperature was determined using a standard Type-B Thermocouple (BAT-24-12, OMEGA Co., Norwalk, CT, USA) for the hot junction and a standard Type-K Thermocouple (XC-14-K, OMEGA Co., Norwalk, CT, USA) for the cold junction. The response time of TFTCs was determined using the drop method. A TFTC sample was placed on a constant-temperature heating table, along with the reference temperature sensor (PT100 Platinum probe, OMEGA Co., Norwalk, CT, USA), and the temperature was set to a constant temperature of 300 °C. A wire connected the output end of the thin-film sample to the data input end of the measuring instrument. When the output voltage and reference temperature were relatively stable, a drop of alcohol was dropped into the sensor’s temperature measurement area. We applied an instantaneous temperature to the sensitive node of the measured high-temperature measurement thin-film, taking advantage of the temperature change caused by alcohol volatilization and heat absorption, with the goal of collecting and recording the thermoelectric potential output time curve of the measured high-temperature measurement thin-film. The thermoelectric characteristic and response drop test systems for tungsten-rhenium TFTCs are schematically depicted in Figure 1b,c.

X-ray diffraction and SEM procedure. For X-ray diffraction (XRD, Rigaku Corporation, Beijing, China, d/max-2400), it had a maximum output power of 3 kW, a rated voltage of 60 kV, and a rated current of 60 mA. A field emission SEM was used to observe the microstructures of the samples (SU-8010, Hitachi Ltd., Tokyo, Japan).

Thermoelectric characteristic calculation (DT). When DT was determined, the hot junction of TFTCs was heated to 1040 °C and the cold junction of TFTCs was kept at room temperature using an ice-water mixture. The heating rate was approximately 5 °C/min. The high-temperature furnace was maintained at 1040 °C for 5 h after the furnace temperature reached the set value. The data logger should be used to determine the change trend in the thermoelectric potential output of the TFTCs and the reference B/K type thermocouple throughout the heat preservation process. The DT ε was set to represent the TFTCs’ stable service time, as shown in Equation (1).

(1) $$\varepsilon \left(T\right)=\frac{△V\left(T\right)}{Vref\left(T\right)}\cdot \frac{T}{△t}\;$$

△V(T) is the voltage drop, Vref(T) is the initial thermoelectric voltage, and △t is the lifetime during which the thermocouple soaked at a particular temperature T for the hot junction.

Thermoelectric characteristic calculation (EMF). When the electromotive force (EMF) behavior of TFTCs was measured, the hot junction was heated to 1040 °C at a rate of approximately 5 °C/min. When the furnace temperature reached the set point, the heating was turned off and natural cooling was activated. Following that, the heating and cooling processes were repeated four times more. EMF behavior was expressed using Equation (2).

*E*(T*) = *A* × (T*)^2^ + *B* × (T*) + *C* (2)

T* refers to the temperature difference between the hot and cold junctions; the unit is °C. *E* denotes the output voltage; the unit is mV. The parameter *C* was arbitrarily set to zero as the boundary condition. T* = 0 must be satisfied in the EMF behavior formula to facilitate the practical application.

## 3. Results and Discussion

### 3.1. Molecular Analysis

**Figure 2:**
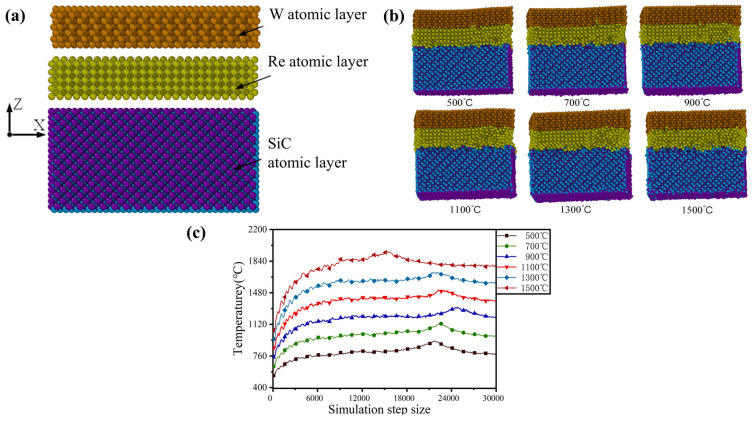
3D molecular dynamics simulation model of silicon carbide and tungsten rhenium metal atomic layer (**a**), diffusion behavior of nano-scale silicon carbide and tungsten (W) and rhenium (Re) atomic layers at different temperatures (**b**), and the temperature changes with the simulation step (**c**) of tungsten-rhenium TFTCs.

We analyze the stability of tungsten rhenium alloys and silicon carbide materials at high temperature through first-principles calculation. Figure 2a,b illustrates the three-dimensional model and diffusion behavior of nanoscale silicon carbide, metal W, and atomic layer Re at various temperatures. Temperatures of 500 °C, 700 °C, 900 °C, 1100 °C, 1300 °C, and 1500 °C were possible. The color of atoms could be determined by their composition. We could see that when the system temperature was maintained above 1100 °C, the increased volume of the atomic layer filled the gap between the atomic layers and the atomic diffusion effect occurred in the gap between the atomic layers. When the temperature exceeded 1300 °C, the atomic diffusion effect between the atomic layers became more pronounced. Equation (3) can be used to express the relationship between the temperature and kinetic energy of a single atom in molecular simulation. Here, *k_B_* is the Boltzmann constant; *T* represents the equivalent temperature of the atom, which can be calculated by the statistical average temperature of the adjacent atoms around the atom; *N* denotes the quantity of atoms in work piece; and instantaneous speed and mass of atom can be expressed by *m_i_* and *v_i_*, respectively.

(3) $$T=\frac{\sum_{i=1}^{N}m_{i}\|v_{i}{}^{2}\|}{3Nk_{B}}$$

Figure 2c illustrates the variation diagrams for temperature with simulation step changes. As can be seen, the average temperature of the model would remain stable as the simulation step was increased against the backdrop of the model’s increased overall temperature. After reaching equilibrium, the average temperature of the model was slightly higher than the temperature set by the system. This is because energy was released during the mutual movement of model atoms, resulting in a slightly higher temperature of the atoms than the temperature set by the system. There were peaks, during which the temperature and kinetic energy increased continuously. During equilibration, the energy of atoms was supplied by the system’s heating, resulting in atomic fractures and the release of energy. Because the model was stable, the number of atoms with broken atomic bonds was also stable, no new energy was released, and the model’s heat dissipation was matched to the system’s temperature during simulation.

**Figure 3:**
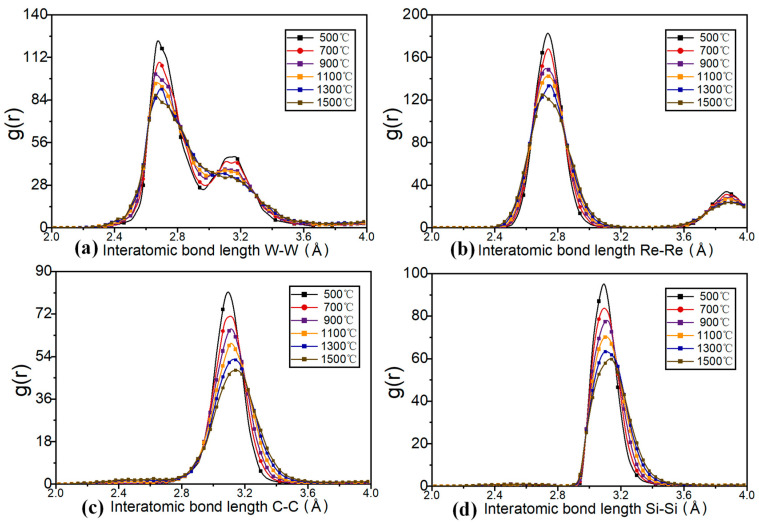
Radial distribution function g(r) among four atomic types W-W (**a**), Re-Re (**b**), carbon-carbon (C-C) (**c**), silicon-silicon (Si-Si) (**d**) of tungsten-rhenium TFTCs.

To gain a better understanding of the work piece’s interatomic growth during heating, the software calculated the work piece’s radial distribution function (RDF) after stabilization. Observing the radial distribution function between four atomic species in Figure 3a–d, the peaks of metal atoms W and Re were 2.7 Å, while the peaks of non-metal atoms C and Si were 3.15 Å. The RDF peaks between metal and non-metal atoms decreased as the temperature increased, owing to the destroyed structure between the atoms. The decrease in atomic spacing and the increase in the non-peak of the RDF function at the peak of the RDF indicated that as the temperature increased, the atomic spacing would expand, resulting in increased damage caused by the model. There was no sharp drop in the peaks of metal atoms as the temperature increased, as is caused by the damaged atomic structure of the outer layer of metal atoms at high temperatures. However, high temperatures did not destroy the internal structure. Due to mutual attraction between atoms, the destroyed metal atoms and non-metal atoms would form a new structure, and the blinding energy of the newly formed bond was less than that of the original lattice structure in the region where the atomic layers were connected.

In general, tungsten-rhenium alloys and silicon carbide materials could be used to design thin-film thermocouples with guaranteed high temperature stability in the 1500 °C range and improved intermolecular thermal stability around 1000 °C.

### 3.2. Size Parameter Design

The thermal stress of the film with different size parameters is calculated, and the suitable size range of the film and substrate is selected through finite element analysis. The compressive thermal stresses between 95% wt. tungsten-5% wt. rhenium (W-5Re) and 74% wt. tungsten-26% wt. rhenium (W-26Re) are listed in Table 2. Table 3 shows the thermal stress between W-5Re, W-26Re films and substrate with different sizes at 900 °C. The results indicated that size parameters had less effect on thermal stress within a certain range, with the maximum thermal stress being 152.7 MPa when the thin-film thickness (D) was 500 nm and the substrate thickness (L) was 10 mm, and the stress was less than the fracture limit. Given the substrate’s strength and convenience of production, the size parameters D = 500 nm and L = 1 mm were chosen.

### 3.3. Thermoelectric and Response Characteristics

**Figure 4:**
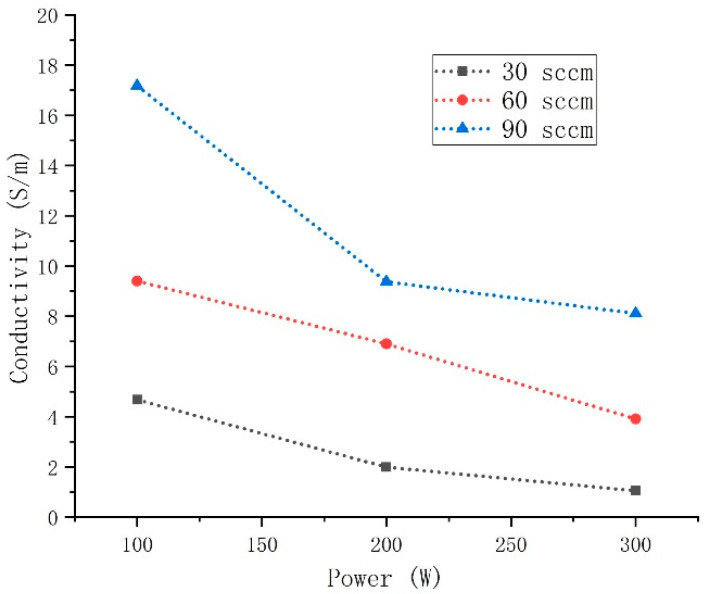
The conductivity of the samples varies with sputtering power and gas flow rate of tungsten-rhenium TFTCs.

As a metallic material, the resistance value of tungsten–rhenium TFTCs increases greatly with temperature, which will affect the impedance matching accuracy in signal acquisition part and increase the test error at high temperature. The film samples prepared with different process parameters were selected to test and compare the conductivity, so as to optimize the process parameter with large conductivity (low resistivity). The conductivity at room temperature was tested, for it is difficult to obtain conductivity of TFTCs at high temperature. Figure 4 illustrates how the conductivity of the samples (Nos. 1 to 9) varied with sputtering power and gas flow rate. Due to the similarity of W-5Re and W-26Re in this experiment, we would discuss only W-5Re here. When the Ar gas flow rate was 90 sccm and the sputtering power was 100 W, the maximum conductivity reached 17.1 S/m, which was approximately 15.2 times greater than the minimum conductivity of 1.05 S/m (30 sccm, 300 W). It was observed that the conductivity of the film decreased with increasing sputtering power and increased with increasing gas flow rate.

**Figure 5:**
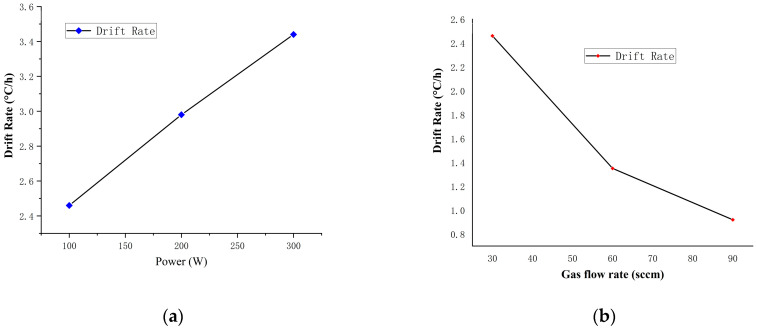
The drift rate (DT) change with power (**a**) and gas flow rate (**b**) of tungsten-rhenium TFTCs samples No. 1, 2, 3, 4 and 7.

In order to obtain a parameter combination with a small DT value while maintaining a large conductivity, we determined the DT of samples prepared using various RF magnetron sputtering parameters, as illustrated in Figure 5a,b. Given the regularity of the change, we fixed the power parameter (100 W for the relatively high conductivity) and increased the gas flow rate from 30 to 90 sccm (samples No. 1, No. 2, and No. 3). When the Ar gas flow rate was 30 sccm and the sputtering power was 100 W, the minimum DT was 0.92 °C/h. Additionally, the gas flow rate was set to 30 sccm and the power was increased from 100 to 300 W (samples No. 1, No. 4, and No. 7). The DT was approximately 3.44 °C/h when the Ar gas flow rate was 30 sccm and the sputtering power was 300 W.

Conductivity decreased with sputtering power and increased with gas flow rate. DT shows an increasing trend with the increase of sputtering power and a decreasing trend with the increase of gas flow rate. For the factor of argon flow rate, the reason for this phenomenon could be that during the sputtering process, Ar gas bombarded the sample’s surface following ionization, causing some ions to escape and thus leading to the conductivity change in tungsten-rhenium TFTCs. The bombardment times increased as the gas flow rate increased, and the metal density and carrier concentration of the film changed. Finally, the thermoelectric characteristics change and affect the DT value. In comparison to the effect of sputtering gas flow rate on conductivity of tungsten-rhenium TFTCs, the effect of sputtering power on conductivity was opposite, which could be due to differences in film formation quality or crystal plane orientation, resulting in a change in the effective mass of the carrier, which has a different impact from gas flow rate. The microstructure was observed to make further analysis.

**Figure 6:**
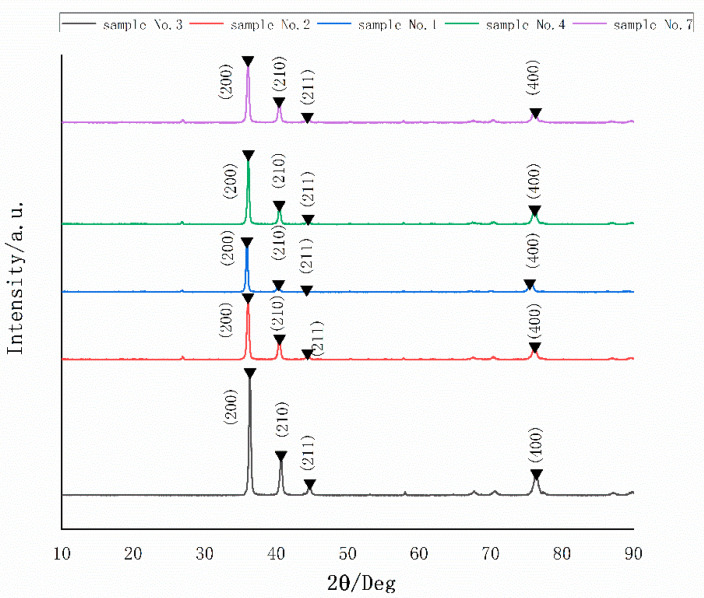
The X-ray diffraction (XRD) patterns for the samples No. 1, 2, 3, 4 and 7.

**Figure 7:**
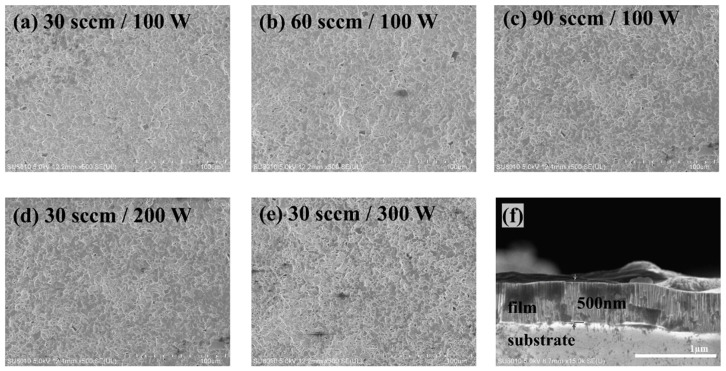
The SEM surface image for samples No. 1 (**a**), No. 2 (**b**), No. 3 (**c**), No. 4 (**d**), No. 7 (**e**) and section image of samples No. 7 (**f**).

Using XRD to analyze the film. The XRD patterns for the samples No. 1, 2, 3, 4 and 7 are shown in Figure 6, which are compared with the XRD standard card (JCPDS card, No.72-1465). The diffraction pattern for the film has some obvious broad peaks at 36.34°, 40.66°, 44.7° and 76.44° corresponding to (220), (210), (211), (400), respectively. It could be observed that the film had a strong (200) preferred orientation. When the power parameter was retained, the diffraction peak intensity increased linearly with the rate of Ar gas flow. Additionally, when the gas flow rate was maintained, the diffraction peak intensity value decreased with increasing power. The increased intensity of the diffraction peak corresponded to an increase in orientation crystallinity, indicating that the total number of (200) orientations was significantly greater than the total number of other crystal faces. In Figure 7a–e, the SEM surface image of the film revealed that certain areas of the surface morphology were connected to the granular structure. The cured films prepared using sputtering technology had similar physical properties in terms of surface appearance, which tended to be dense and flat. The SEM Section image demonstrates that the films had the same thickness as expected for the preparation described in Figure 7f and were composed of small particles with diameters oriented vertically and randomly distributed on the surface. Given the similarity between W-5Re and W-26Re, we selected W-5Re as the representative.

Moreover, if conductivity decreased with sputtering power and increased with gas flow rate while DT increased with sputtering power and decreased with gas flow, it could be assumed that conductivity and DT had an inverse relationship. However, at this point, there was no theoretical model to explain. Additionally, the parameter above would not be the only factor affecting the conductivity and DT of the tungsten-rhenium TFTCs, as different substrate temperatures could affect them. The substrate temperature was held constant at 100 °C in these experiments.

**Figure 8:**
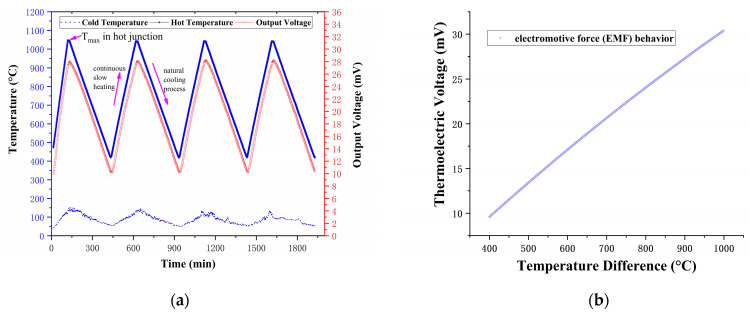
The thermoelectric performance of tungsten-rhenium TFTCs in four heating cycles of 33 h (**a**) and the electromotive force (EMF) behavior of TFTCs (**b**).

Accordingly, additional performance testing was prepared under this parameter. The sample was sputtered with an Ar gas flow rate of 30 sccm and a sputtering power of 100 W (as No. 1) to determine its thermoelectric performance. The repeatability of the TFTCs was determined over a 33 h period using four heating cycles. Figure 8a,b, Table 4, and Equation (4) illustrate the results.

*E*(T*) = −7.562 × 10^−6^ × (T*)^2^ + 0.0236 × (T*) (4)

**Figure 9:**
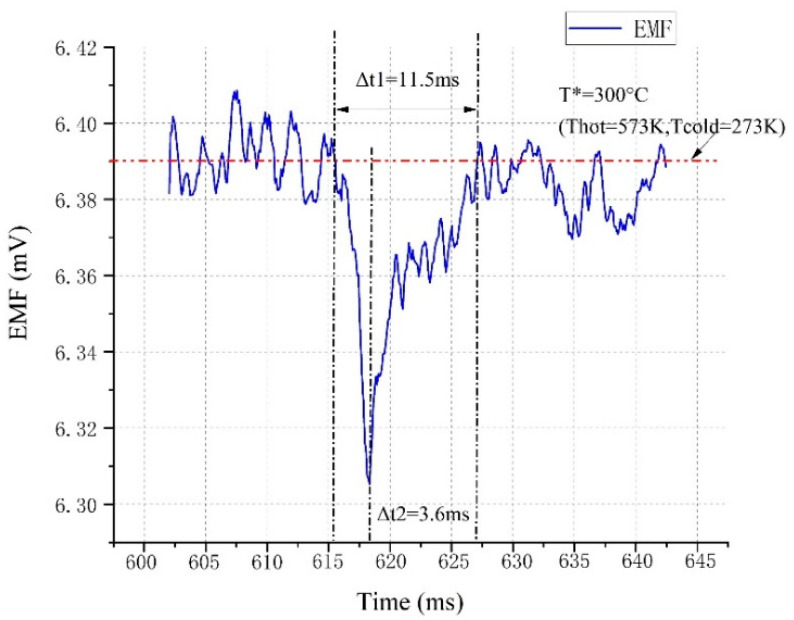
Response drop test result of TFTCs.

The three cycles had a maximum standard deviation of 0.428 mV. The tungsten-rhenium TFTCs had a repeatability error of ±1.37%. The peak thermoelectric voltage of the TFTCs was 28.0 mV when the temperature difference between the hot and cold junctions was 900 °C (the hot junction temperature was 1040 °C). The average Seebeck coefficient reached 31.1 µV/°C at 900 °C temperature difference, 89.6% larger than the average Seebeck coefficient of the standard C-type (tungsten-rhenium, 16.40 µV/°C at 900 °C [19]) for the component difference in RF magnetron sputtering between the metal wire and film. Furthermore, the lumped capacity method test indicated that the response time was 11.5 ms when the temperature was maintained around 300 °C, as illustrated in Figure 9.

## 4. Conclusions

Tungsten-rhenium TFTCs exhibited an average Seebeck coefficient of 31.1 µV/°C at 900 °C temperature difference (hot junction 1040 °C) with a repeatability error at ±1.37% in 33 h. It was observed that Tungsten-rhenium TFTCs with a conductivity of 17.1 S/m exhibited the highest thermoelectric stability. The DT reached 0.92 °C/h (9.5% of the old type tungsten-rhenium TFTCs we made) in 5 h of service period at 1040 °C, which is 4.5% of current ITO vs. In_2_O_3_ TFTCs (ITO 90%/In_2_O_3_ 10%, 1200 °C with a DT of 20.4 °C/h [20]). In addition, the lumped capacity method test shows that the response time is 11.5 ms at 300 °C. For tungsten-rhenium TFTCs, the conductivity decreased with the increase in the sputtering power, and increased with the Ar gas flow rate. The DT significantly decreased when the sputtering power increased, which indicated the improvement of thermal stability at high temperatures. This improves the high temperature stability of the existing tungsten-rhenium TFTCs, which show that the high thermal stability was found with a high significance in real-time temperature measurement for narrow spaces such as an aero-engine combustion chamber.

## Figures and Tables

**Table 1:** Sputtering parameters of W-5Re, W-26Re thin-films.

Sputtering Parameters	Samples								
	No. 1	No. 2	No. 3	No. 4	No. 5	No. 6	No. 7	No. 8	No. 9
Gas flow rate (sccm)	30	60	90	30	60	90	30	60	90
Power (W)	100	100	100	200	200	200	300	300	300

**Table 2:** The material properties of W-5Re, W-26Re and substrate.

Materials	Poisson’s Ratio	Young’s Modulus (GPa)	Coefficient of Thermal Expansion (10^−6^/K)
silicon carbide ceramic	0.29	390	7.7
95% wt tungsten—5% wt rhenium	0.281	362	4.61
74% wt tungsten—26% wt rhenium	0.285	398	5.03

**Table 3:** The thermal stress between W-5Re, W-26Re films and substrate with different sizes at 900 °C.

D (µm)	L (mm)	Thermal Stress between W-5Re and W-26Re (MPa)	Thermal Stress between W-5Re and Substrate (MPa)	Thermal Stress between W-26Re and Substrate (MPa)
0.5	10	43.5	152.7	131.9
0.5	5	43.3	152.3	131.7
0.5	1	43.1	151.9	131.4
2	1	42.9	151.3	130.9
10	1	41.6	146.3	126.9

**Table 4:** T Polynomials coefficients of the TFTCs.

A (mV/°C^2^)	B (mV/°C)	C (mV)	Average Seebeck Coefficient at 900 °C (µV/°C)	Average Seebeck Coefficient of Standard C-Type Thermocouple 900 °C (µV/°C)
−7.56 × 10^−6^	0.023	0	31.1	16.4

## Data Availability

The data is available on reasonable request from the corresponding author.
